# A mixed-methods study of community dog management and rabies awareness in Thailand

**DOI:** 10.1016/j.onehlt.2026.101596

**Published:** 2026-09-28

**Authors:** Weerakorn Thichumpa, Anuwat Wiratsudakul, Wirichada Pan-ngum, Thomas Rawson, Katharina D. Hauck, Tini Garske

**Affiliations:** a MRC Centre for Global Infectious Disease Analysis, Jameel Institute, School of Public Health, Imperial College London, London, UK; b Department of Clinical Sciences and Public Health, and the Monitoring and Surveillance Center for Zoonotic Diseases in Wildlife and Exotic Animals, Faculty of Veterinary Science, Mahidol University, Nakhon Pathom, Thailand; c Mahidol Oxford Tropical Medicine Research Unit (MORU), Faculty of Tropical Medicine, Mahidol University, Bangkok, Thailand; d Department of Tropical Hygiene, Faculty of Tropical Medicine, Mahidol University, Bangkok, Thailand; e Leverhulme Centre for Demographic Science, Nuffield Department of Population Health, University of Oxford, Oxford, UK; f MRC Centre for Global Infectious Disease Analysis, School of Public Health, Imperial College London, London, UK

**Keywords:** Community dogs, Rabies, Vaccine, Sterilization, Mixed methods, One health

## Abstract

Rabies elimination requires coordinated interventions across the human-animal-environment interface, yet sustained progress is often hindered by systemic gaps between dog population management, community engagement, and governance. This study investigated community perceptions and challenges related to rabies prevention and control across high-endemic areas in Thailand. Using a mixed-methods design, secondary provincial quantitative data were integrated with qualitative thematic analysis from 57 stakeholders in high endemic areas, including public health officials, policymakers, NGOs, animal-welfare liaisons, and community members. The findings indicated standard rabies awareness, 98.2% of participants demonstrated good preventive practices, recognized post-exposure prophylaxis (PEP) as essential, and 68.4% possessed good knowledge. Although most supported dog sterilization (59.6%) and vaccination (63.2%), only 19.3% perceived existing control campaigns as effective. Main challenges included constrained operational resources (57.9%) and limited local government budgets (40.4%). Free-roaming community dogs were prevalent, with 54.4% of participants identifying abandonment as the primary driver of population growth. Informal feeding networks provided localized care for community dogs. While public willingness to fund management was relatively low (33.3% willing to pay; 19.3% supporting co-payment), reflecting an expectation of local authority responsibility. Qualitatively, unclear boundaries between public health mandates and animal welfare legal frameworks added complexity to rabies outbreak response and sometimes introduced institutional challenges among local government and non-governmental sectors. Nevertheless, stakeholders strongly supported sustained sterilization and intersectoral collaboration. Addressing policy-practice gaps, stabilizing financing, and integrating community social dynamics into formal One Health governance are crucial for achieving sustainable rabies elimination.

## 1 Introduction

Rabies continues to threaten both human and animal health worldwide, with the majority of human rabies cases resulting from dog-mediated transmission [1], [2]. In Thailand, dogs are the main reservoir and source of rabies transmission, and despite sustained national efforts through mass dog vaccination campaigns, human rabies cases are still reported annually [3], [4], [5], [6], [7], [8]. Free-roaming dogs are generally owned or regularly fed within residential areas but are allowed to roam freely and often receive diffuse care from multiple households. In contrast, unowned dogs lack guardianship and survive independently [9], [10]. Both groups function as “community dogs”, whose unrestricted movement and diffuse ownership hinder systematic vaccination and sterilization [7], [8], [9], [10], [11].

In high-prevalence areas of Thailand, public awareness of rabies prevention and control remains limited [8]. Effective implementation of dog management programs requires an analysis of the socio-economic and logistical factors that limit the uptake of sustained vaccination and sterilization initiatives. In addition, empirical evidence on community engagement, attitudes, ownership practices, and perceptions of rabies risk can further enhance the design and success of context-specific interventions [8], [9], [12]. Effective dog population management and rabies prevention can be advanced through integrated One Health approaches that combine field-based data collection with community engagement and systematic evaluation of interventions, generating actionable evidence to support sustainable, locally appropriate strategies.

Our previous modeling and economic analyses aligned with the World Health Organization (WHO) and the World Organisation for Animal Health (WOAH) guidelines that support a 70% dog vaccination with female sterilization as a cost-effective strategy to control rabies and population growth [1], [4], [5], [11], [13]. Yet, translating these findings into practice requires navigating social, cultural, and institutional contexts. Because of limited budgets and resources, vaccination coverage may be lower in some areas where rabies cases are rarely reported. This situation contributes to variability in vaccination coverage across the provincial level. Additionally, effective dog population management and rabies prevention cannot be achieved through veterinary or public health interventions alone, they require cross-sector collaboration and active community engagement. An understanding of local perceptions and practices is therefore essential to bridge the gap between policy formulation and effective implementation. Complementing previous analytical and modeling studies [7], [8], [11], this study integrated social epidemiology using a mixed-methods approach to examine socio-economic factors, practical constraints, and accessibility issues affecting dog population management and rabies prevention efforts. The findings aim to support information to policy-making consideration and support Thailand's national goal of rabies elimination by 2030.

## 2 Methods

### 2.1 Study sites and participants

The qualitative interviews and surveys were conducted in Thailand, between October 2025 and January 2026. Chonburi (eastern region) and Songkhla (southern region) provinces were selected as study sites due to their history of recurrent annual rabies outbreaks in both dogs and humans [3], [4], [6], [8], [14], [15] (Fig. 1).

**Fig. 1:**
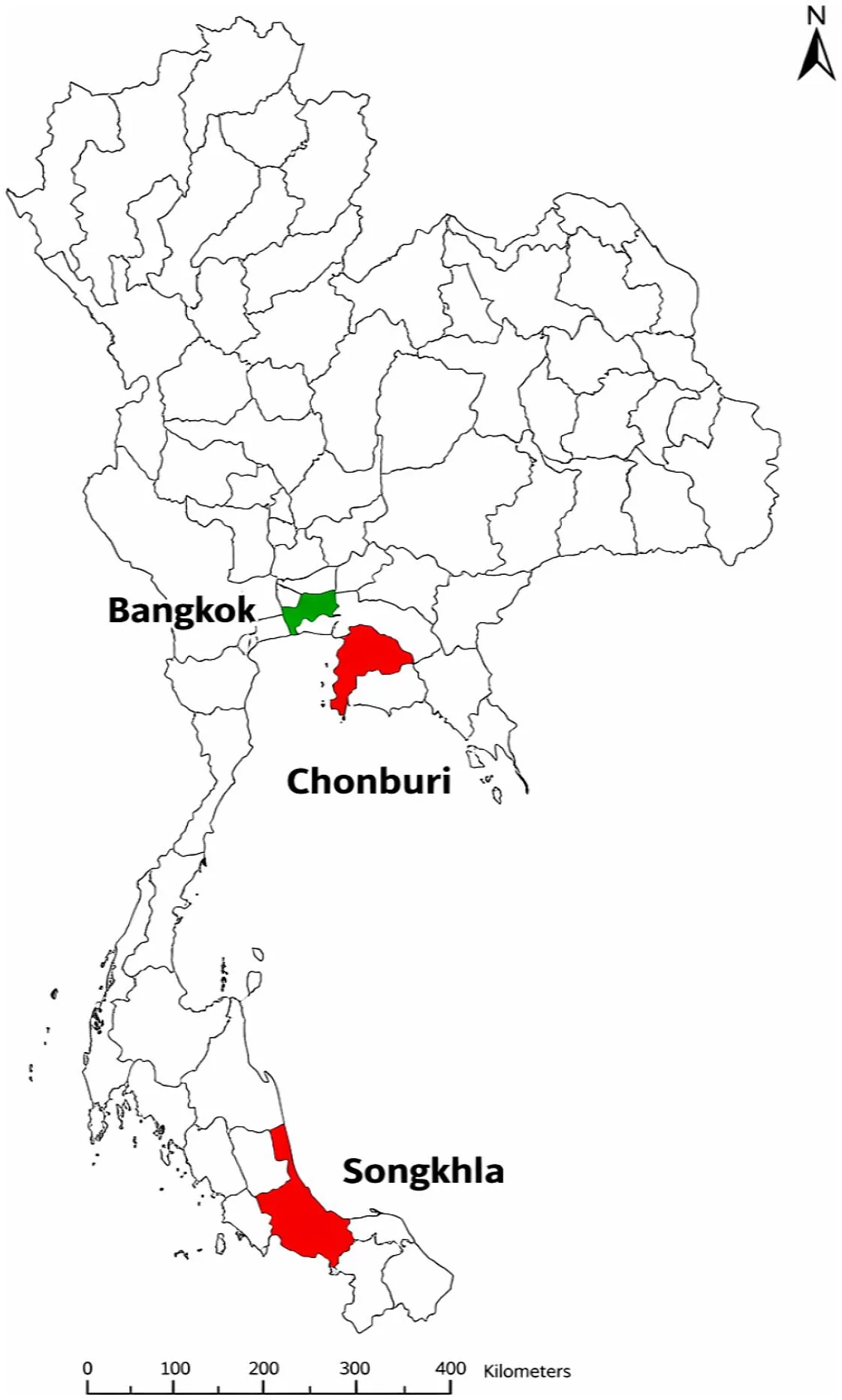
Map of study sites in Thailand: Chonburi (eastern region) and Songkhla (southern region), with recurrent annual rabies outbreaks in both dogs and humans. Bangkok is shown as the national headquarters for dog population management and rabies prevention.

Participants were purposively sampled across provincial, district, and local levels through official veterinary, public health, and government organizations. Overall, participants aged 18 years and older participated, including veterinarians, animal liaisons, public health liaisons, policy makers, practicians, dog owners, and community members. Recruitment was based on participants' direct roles and expertise in community dog management and rabies prevention, as well as community members living in close contact with community dogs. All recruitments were conducted through formal organizational and community channels. Following a qualitative method, no formal sample size calculation was performed in this study, instead, the study emphasized capturing diverse perspectives and in-depth insights [16].

### 2.2 Data collection

Data was collected through semi-structured interview form and structured surveys designed to capture both quantitative and qualitative information (Supplementary information 1). Face-to-face interviews were conducted with participants from multiple sectors to obtain in-depth information and perspectives. Primary data focused on the characteristics of community dogs (e.g., living areas, population size, vaccination, and sterilization) and the rabies awareness aspect (e.g., knowledge, prevention practices, attitude to severity, rabies situation and prevention campaigns) among stakeholders. We defined community dogs as both free-roaming owned dogs and unowned dogs that live around community areas or remain in close contact with humans (e.g., local feeders, community members, or general public). Secondary quantitative data were obtained from official governmental reports and local administrative records among provincial and district levels [3], [4], [6], [14], [15], [17], [18], [19], [20]. These included estimates of the number of dogs, laboratory-confirmed rabies cases, and vaccination and sterilization coverage in dogs. Human-related data included the use of rabies post-exposure prophylaxis (PEP), which served as an important indicator of dog-bite incidents and rabies risk prevention [21], [22], [23]. Collectively, these data provide epidemiological context for describing rabies risk and ongoing situation in the study areas. Qualitative data were derived from interviews and survey responses, exploring themes related to dog population management, vaccination practices, rabies awareness, prevention strategies and related cost aspects.

### 2.3 Data analysis

A mixed-methods approach was used to integrate quantitative and qualitative data, providing a comprehensive assessment of community dog management and the rabies control landscape. Survey data were analyzed using descriptive statistics, with frequencies and percentages calculated for participant demographics and key perspectives. Data from in-depth interviews were analyzed using thematic analysis. This process involved iterative stages of coding and categorization to identify patterns in participants' narratives, guided by existing public health frameworks on dog population management and rabies prevention. The integrated analysis focused on four key themes: rabies knowledge and awareness, community dog management and attitudes, policy and governance aspects, and economic considerations relevant to sustainable program implementation. Given that key stakeholders frequently fulfilled multiple concurrent social roles within the community (e.g., local feeders, community health volunteers, dog owners, etc.), survey findings were analyzed as an aggregated dataset to reflect holistic perspectives without introducing role-assignment confounding.

## 3 Results

### 3.1 Participant characteristics

A total of 57 participants were included, comprising 28 from Songkhla and 29 from Chonburi provinces. Most participants were aged 50–59 years (50.9%), followed by 60–69 years (28.1%). The minor proportions were aged 40–49 years (8.8%) and ≥70 years (8.8%), while only 3.5% were aged 30–39 years. Participants aged ≥70 years were observed only in Chonburi. The age distribution ranged from 30 to 79 years, with a mean age of 57 years across both provinces.

Business owners represented the largest occupational group (36.8%), followed by government officers (22.8%) and housekeepers or retired individuals (19.3%). General laborers or freelance workers accounted for 15.8%, and other occupations were 5.3%. The majority of participants were dog owners or dog carers (71.9%). Participants frequently reported multiple community roles. The most common role were local dog feeders (63.2%), followed by community supporters, e.g., volunteers, donators, campaign supporters, dog surveyors and reporters (36.8%) and public health volunteers (31.6%). Other roles included livestock volunteers or liaisons (14.0%), community leaders (12.3%), policy makers or practitioners (12.3%), animal welfare advocates (10.5%), and Non-Governmental Organization (NGO) founders for dog and animal welfare (5.3%) (Table 1).

**Table 1:** Participant characteristics (*N* = 57).

Characteristics	Categories	Songkhla (*n* = 28)	Chonburi (*n* = 29)	Total (*N* = 57)	Percentage (N = 57)
Age (years)	30–39	2	0	2	3.51
	40–49	5	0	5	8.77
	50–59	15	14	29	50.88
	60–69	6	10	16	28.07
	70+	0	5	5	8.77
	Mean age	52	61	57	
	Age range (30–79 years)				
Primary occupation	Business owner	12	9	21	36.84
	Government officer	7	6	13	22.81
	Housekeeper or retried	4	7	11	19.30
	General Laborer or freelance	4	5	9	15.79
	Others	1	2	3	5.26
Dog relationship	Personal owner	19	22	41	71.93
	Not owner	9	7	16	28.07
Community Roles	Local feeders	22	14	36	63.16
(Multiple responses)	Community supporters	12	9	21	36.84
	Public health volunteers	4	14	18	31.58
	Livestock volunteers or liaisons	4	4	8	14.04
	Community leader	3	4	7	12.28
	Animal welfare advocate	4	2	6	10.53
	Policy maker or practitioner	3	4	7	12.28
	NGO founder or animal foundation	3	0	3	5.26

### 3.2 Local reports and secondary information

According to the local government reporting systems in Songkhla and Chonburi provinces [3], [4], [6], [14], [17], [20], the estimated dog population was reported. In Songkhla, the reported estimated number of dogs increased from 90,000 in 2021 to a projected 140,000–180,000 dogs by 2025 [14], [20]. A similar trajectory was observed in Chonburi, where the population expanded from 200,000 to an estimated 310,000–420,000 dogs over the same period [14], [17]. These findings indicate a rapid expansion of the dog population in both provinces. The summarized information is shown in (Fig. 2).

**Fig. 2:**
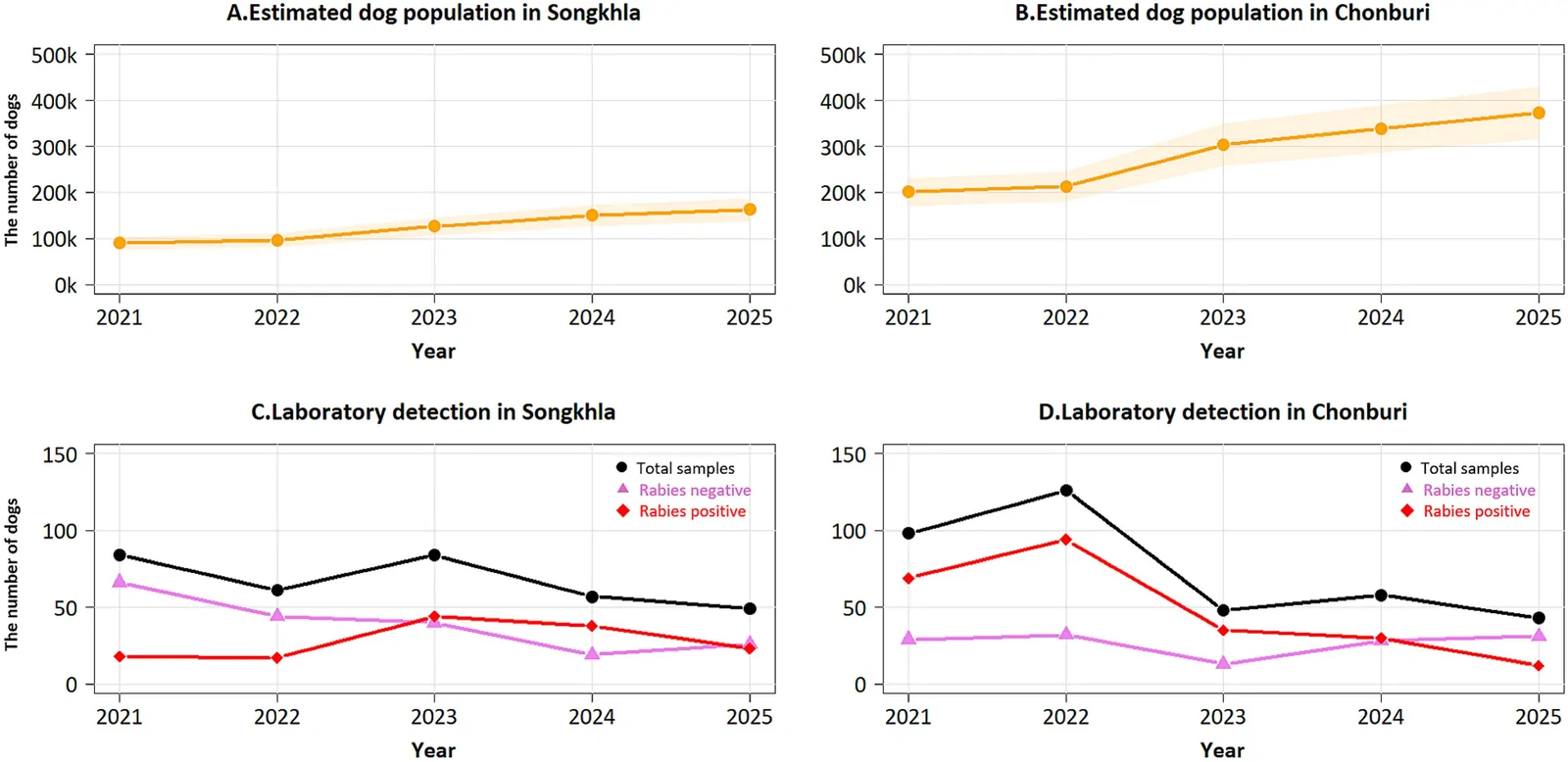
(A-B) Provincial monitoring of estimated dog population size [[14], [17], [20]], and (C-D) Reported dog rabies by laboratory detection in Chonburi and Songkhla provinces [4], [6], Thailand, between 2021 and 2025.

Due to the limited availability of longitudinal records, the annual number of dog sterilizations was estimated using a resource-based approach by the Provincial Livestock Offices. In Songkhla, sterilization capacity averaged approximately 5000 cases per year (range: 4500-5500), while in Chonburi the average was around 6500 cases annually (range: 5000-8000) [17], [20]. These estimates were derived by cross-referencing annual fiscal budgets with sterilization quotas and existing personnel resources allocated to local administrative organizations at the sub-district levels. Based on these estimates, sterilization coverage appeared relatively limited compared with the overall size of the dog population. As the dog population increased over time, the proportional coverage of sterilization was estimated to decline to approximately 1–2% of the total dog population by 2025.

For dog rabies vaccination, the Provincial Livestock Offices targeted a standard coverage of 70% annually. Budget constraints led vaccination campaigns to prioritize endemic and high-risk areas, resulting in variation in vaccination numbers across districts and sub-districts in both provinces. In Chonburi, the Provincial Livestock Office reported an annual range of approximately 15,000–23,000 vaccine doses per district [17], while in Songkhla the annual range was about 9000–15,000 doses per district [20].

National reports recorded four and six human rabies deaths in Songkhla and Chonburi, respectively, between 2017 and 2024 [18]. Reported confirmed laboratory detections of dog rabies in both provinces declined by 2025 (Fig. 2C-D). However, this trend should be interpreted with appropriate caution, as it coincided with a substantial reduction in the number of samples submitted for laboratory testing. In Songkhla, 18 positive cases were detected from 84 samples (21%) in 2024, compared with 23 positive cases from 49 samples (46%) in 2025. In Chonburi, the proportion declined from 69 positive cases out of 98 samples (51%) in 2024 to 12 positive cases out of 43 samples (28%) in 2025 [4], [6], [17], [20]. With fewer tests conducted overall, some of the observed declines may relate to reduced surveillance rather than actual changes in disease occurrence.

For human PEP, the estimated number of treatments remained high in both provinces, exceeding 20,000 cases annually during 2021–2025. The estimates were performed from provincial health office records and annual vaccination reports from selected hospitals [17], [20]. Large provincial or tertiary hospitals typically reported approximately 4000–6000 individuals receiving PEP each year, whereas community, medium-sized, or private hospitals generally reported between 100 and 800 cases annually [17], [20], [24]. Considering that each province has roughly five large hospitals and more than twenty small-and-medium or private hospitals providing rabies vaccination services, the total number of annual PEP treatments was estimated to >20,000 cases. We interpreted the approximate estimations as reporting coverage varied across hospitals and the available data did not include all government and private hospitals in both provinces. Nevertheless, the consistently high number of PEP treatments suggests sustained demand for rabies exposure management services and indicates a persistently high level of animal-bite exposures in the population, most associated with dog bites. Summary details are provided in Supplementary information 2.

### 3.3 Attitude surveys

#### 3.3.1 Rabies knowledge and awareness

Knowledge and practice were assessed using three key indicators: mammalian hosts, immediate wound management (washing with running water and soap for 15 min), and adherence to PEP protocols following a bite. Attitude was evaluated in terms of perceptions of rabies severity, emphasizing whether it was regarded as a critical or life-threatening concern. Overall, participants demonstrated correct knowledge about rabies prevention. Most participants (98.2%) reported good preventive practices after getting bitten or risk exposure, such as wound dressing, seeking medical treatment, and received vaccination. In addition, 68.4% demonstrated good general knowledge about rabies, including understanding that it is a fatal disease and mainly transmitted through dog bites and infected mammals. More than half of the participants (54.4%) were aware of rabies prevention activities and vaccination campaigns in their area. Participants who perceived rabies as not a severe issue in their community (66.7%) explained that they had never found any rabies cases. One participant remarked, “It's not severe. We have good protection, and vaccination coverage is quite extensive. People who are bitten can receive vaccination and treatment at the hospital.” Similarly, one medical practitioner commented, “Rabies outbreaks in this area are rare and have been absent for a long time.”

However, 33.3% clearly expressed strong concern about the severity of rabies and 26.3% had experiences with rabies cases in humans and animals. Participants noted the presence of many free-roaming dogs in their communities (78.9%), often living together in large groups. They expressed concern that rabies could become a serious threat if these dogs remained uncontrolled, unvaccinated, and difficult to catch. One participant reported, “Four to five months ago, a rabid dog came into my house and died three days later. I worry about the high risk to my dogs and cats.” Another commented from a health volunteer, “It's very severe, there is no population control, no vaccinations, and the dogs form in large groups. People who feed them ignore prevention programs.” A veterinarian added, “The disease is severe, and villagers lack knowledge and awareness. I once encountered a dog brought to the clinic for treatment that later died. After detection, it was a rabies case” (Table 2).

**Table 2:** Thematic findings (N = 57).

Themes	Key codes	n	%	Description
Rabies knowledge and awareness				
	Good practice to prevention	56	98.2	Knowledge of standard practice (e.g., washing wounds, PEP)
	Good rabies knowledge	39	68.4	Knowledge of rabies transmission, risks, and prevention
	Awareness of rabies prevention and campaigns	31	54.4	Recognition of existing public health campaigns and activities
	Attitude to rabies severity	19	33.3	Perception of rabies is a critical or life-threatening threat
	Experiences with human or animal rabies	15	26.3	Personal or community history with actual infection cases
Community dog management and attitude				
	Negative impact of dogs on community	46	80.7	Concerns regarding noise, waste, aggression, or perceived danger
	Wide distribution of free-roaming dogs	45	78.9	High populations in public (e.g., roads, temples, forests, camps, landfills)
	Local dog feeders as primary carers	37	64.9	Feeders are seen as caretakers or the ones in charge
	Increased dogs due to abandonment	31	54.4	Identifying irresponsible ownership as a cause of rising populations
	Feeders caring for community dogs	27	47.4	Feeders supply food and basic care as available
	Owner responsibility for dogs	27	47.4	Belief in the duty of individuals to manage their own animals
	Human-dog relationship support	25	43.9	Perceptions of dogs as parts of mental well-being and happiness
Policy and governance aspect				
	Vaccination campaigns prevent rabies	36	63.2	Viewing rabies vaccine as a key preventive measure
	Population control through sterilization	34	59.6	Viewing sterilization as a key for population control
	Limited practitioners and human resources	33	57.9	Viewing campaigns as constrained by limited human resources
	Local announcements and campaign notification	31	54.4	Conduct campaigns via community communication channels
	Limited local government budget support	23	40.4	Viewing insufficient budget hinders program success
	Sterilization insufficiency	23	40.4	Perceiving sterilization is not enough to control dog populations
	Vaccine insufficiency	15	26.3	Perceiving dog vaccination coverage in communities as insufficient
	Success of campaigns	11	19.3	Perceiving current intervention as successful
	Multi-sector involvement	4	7.0	Mentions of multi-sectoral collaboration in current interventions
Cost aspect				
	Economic capacity influences payment decisions	43	75.4	Household income is the primary barrier to accessing services
	Government responsibility for community dogs	19	33.3	Perceiving community dogs should be managed by government
	Willingness to pay	19	33.3	Participants expressing willingness to pay for dog-related services
	Co-payment by government and community	11	19.3	Expressing preference for co-payment for dog-related services

#### 3.3.2 Community dog management and issues

Free-roaming dogs were commonly seen throughout the community. Approximately 78.9% of participants reported that community dogs were widely distributed across public and communal areas, including roads, temples, schools, community forests, graveyards, and other abandoned zones. Most of them (80.7%) perceived dogs as having negative impacts on the community, including concerns about safety, noise, accidents, waste accumulation, poor sanitation and hygiene. A public health staff also mentioned “We have many problems. Some people complain a lot. They worry about accidents, and dogs bite people. Many dogs stay around because someone feeds them. Some owners don't know what to do, so when they cannot take care of the dog, they just abandon it.” While informal care of those community dogs was common. Overall, 64.9% identified local dog feeders as the primary caregivers of community dogs. Among this group, nearly half (47.4%) reported that feeders routinely provide food and basic health care. In addition, 54.4% believed that the increasing number of dogs was related to abandonment by owners. An NGO member said, “It really has to start with raising awareness. When people own their dogs, they need the right knowledge, and the rules have to be enforced to stop abandonment. Otherwise, it causes problems for everyone. Owners just leave their dogs or let them roam around, and it becomes an issue in the community.” Regarding responsibility, 47.4% emphasized that dog owners should be responsible for their dogs. Interestingly, 43.9% mentioned positive aspects of human-dog relationships, such as emotional support and companionship, suggesting mixed attitudes toward community dogs (Table 2).

#### 3.3.3 Policy and governance

Vaccination and sterilization were generally recognized as important strategies. About 63.2% agreed that vaccination campaigns were effective in preventing rabies, while 59.6% supported sterilization as a method for population control. However, only 19.3% mentioned the success of dog population control and rabies prevention campaigns in their communities. Several implementation-related challenges and limitations were identified. More than half of participants (57.9%) mentioned limited practitioners and human resources as an important key. Also, around 40.4% indicated that local government budgets were insufficient and sterilization efforts were not enough to control the dog population. Vaccination coverage insufficiency was also mentioned by 26.3% of participants. Although 54.4% were aware of local announcements and campaign notifications, only 7.0% referred to multi-sector collaboration, suggesting limited cross-sector involvement in rabies control activities. A participant mentioned that “You can't really run proper rabies campaigns without the money to support. When the funds are limited and there aren't enough staff, I feel that the campaign has a hard chance to succeed eventually.” While one of community supporter mentioned “In my community, people are willing to participate and support the campaigns. But when they brought or caught their dogs for vaccination and sterilization, the resources weren't enough, and their dogs didn't get sterilized. I'm afraid that if people feel disappointed, they might be less willing to come again in the future” (Table 2).

#### 3.3.4 Cost aspect

Financial factors influenced participation in rabies prevention programs. About 75.4% reported that economic capacity affected their ability to provide vaccine, sterilization, and animal health care programs. Only 33.3% of the participants reported that they were willing to pay for animal health services for their dogs. In addition, 19.3% preferred a co-payment system shared between the community members and the government. For community dogs, most participants indicated that they were not willing to pay for vaccination or sterilization services. In addition, 33.3% stated that the government should take primary responsibility and allocate sufficient funding for community dog management and rabies prevention programs. Dog owners should allocate an appropriate budget for the care and management of their pets (Table 2).

## 4 Discussion

In Songkhla, local reports indicated that both the dog population and rabies-positive detections increased annually, reaching their high levels in 2024 [6], [14], [20]. Chonburi reported a rise in the dog population, while laboratory-confirmed rabies cases declined [6], [14], [17]. The expanding dog population and fluctuating rabies positive detections, combined with sustained demand for human PEP, indicated that dog-bite exposure remains a persistent public health concern. In addition, the participants were relatively older (mean age 57 years; range 30–79). Ensuring the long-term sustainability of rabies management may benefit from engagement of younger community members. Strengthening age diversity and community participation will help sustain local surveillance and support networks, thereby ensuring the long-term continuity of rabies control efforts [1], [25].

Qualitative findings support the secondary information and epidemiological trends. A steady increase in dog abandonment (noted by 54.4% of participants), paired with a limited annual sterilization capacity (5000–6000 dogs/year), has driven rapid dog population growth. Regarding local budget constraints (reported by 40.4% of stakeholders), effective sterilization coverage fell to 1–2% by 2025. This low rate leaves current interventions insufficient to stabilize the dog population [17], [20]. Few participants viewed rabies as severe due to the absence of recent local deaths, aligning with our previous findings that rabies severity is routinely minimized by personal ignorance and passive attitudes [7], [26]. However, secondary data revealed high annual PEP demand exceeding 20,000 cases (>14 per 1000 population) [17], [20]. This discrepancy confirms that risk perception is driven by visible mortality rather than actual transmission risk, sustaining dependence on costly reactive PEP over proactive dog control.

Rapid dog population growth has widened the control gap, exceeding the fixed capacity of local sterilization, vaccination, and surveillance systems. Between 2021 and 2025, estimated dog populations nearly doubled while annual sterilization quotas remained static due to fixed budgets. Although vaccination targets aligned with WHO/WOAH recommendations, resource constraints caused uneven coverage and potential immunity gaps. Note that these estimates reflect state-funded activities only, as NGOs and private veterinary services were unrecorded. Furthermore, frequent administrative turnover and shifting local policy priorities disrupt program continuity, hindering long-term rabies control [11]. The limited resources and budgets, and lack of surveillance capacity have constrained rabies prevention [27], [28]. Similarly, our findings indicated that constrained resources and fixed annual budgets limit the scale and consistency of vaccination and sterilization campaigns. These financial and operational barriers restrict personnel, logistics, and supplies, while inadequate surveillance hinders case detection, outbreak investigation, and evaluation. Overall, these constraints may have compromised the long-term sustainability of rabies control. A more flexible strategy with stable funding is essential to reduce transmission risks and control population growth [29], [30].

Additionally, the sustained high number of human PEP treatments indicated continued risk of rabies exposure and dog bites. The general dog-bite rate has been reported at approximately 7 bites per 1000 population [31]. Nationally, an estimated 286,000 PEP courses were administered in 2024, equivalent to roughly 4 treatments per 1000 people [19]. However, the estimated annual PEP numbers in our study areas exceeded 20,000 cases [17], [20], [24], representing approximately 14 PEP treatments per 1000 population. Although site-specific billing data were unavailable, the local report estimated a full course of PEP costs roughly $40–$100 USD per bite [20]. The results highlighted an ongoing, systemic public health challenge. Despite the use of both active and passive population control measures and mass vaccination, rapid dog population growth continued. Likewise, the increasing dog biting rate, together with the resulting demand for medical care, has imposed a persistent and substantial economic burden on the healthcare system [32], [33]. The sustained demand for PEP demonstrated that dog bites were an ongoing socio-environmental challenge. High bite incidence further highlighted that dog vaccination and population control efforts had remained insufficient. As urban areas expanded and human populations grew, human–dog interactions increased, maintaining high PEP demand unless animal-level controls had been strengthened. The situation underscored the need for accessible healthcare services capable of providing timely PEP to exposed individuals and highlighted the broader One Health imperative [13], [34], [35], [36].

Participants demonstrated strong practical knowledge of post-exposure procedures, yet 68.4% described rabies as non-severe. This low-risk perception was primarily shaped by the prolonged absence of local human fatalities, leading stakeholders to evaluate rabies risk based on visible outcomes rather than ongoing transmission potential. Consequently, strong individual motivation for PEP failed to translate into collective engagement for sustained dog population management or surveillance. Individual awareness for PEP was strong, however, collective engagement in sustained population management and surveillance appeared less consistent. Unless rabies is addressed as an intersectoral threat, reliance on PEP will continue shifting the burden onto human healthcare rather than stopping transmission at the animal source. Overall, the findings suggested that while reactive knowledge was strong, proactive risk awareness and sustained preventive engagement required further strengthening to reduce vulnerability to future outbreaks. To address this gap, strengthened health education programs should be implemented to reinforce understanding of rabies severity and its public health impact [8], [37]. Regular updates on local rabies activity from authorities are essential to sustain public vigilance, reinforce preventive behaviors, and support long-term disease monitoring.

Community dog management operated through a combination of safety concerns, informal caregiving, and social dynamics. Although health and safety concerns were most common, some participants valued community dogs for companionship. They noted that aggressive behavior usually results from mistreatment, which makes these dogs harder to catch for vaccination and sterilization programs. Continuous abandonment at public places and construction sites, alongside high birth rates, leads to rapid dog population growth. Most participants agreed that irresponsible ownership, rather than the dogs themselves, is the primary issue. Routine feeding was reported as practical, keeping community dogs localized and less disruptive. However, many emphasized that feeding alone was insufficient for sustainable management, particularly given the financial burden of long-term informal care. While feeding practices did contribute to animal welfare, they often operated separately from formal governmental campaigns. This pattern reflected a governance gap, in which responsibility for dog management shifted from individual owners to the wider community. The link between dog abandonment and population growth demonstrated weak owner accountability. Addressing this requires a combination of public education and stricter regulation. Systematic and continuous sterilization was considered essential for controlling population growth. This coexistence of concern and attachment suggested that community-dog issues could not be addressed solely through control measures but required socially sensitive and context-specific strategies [1], [13], [35], [38].

Despite positive attitudes toward sterilization and vaccination, few participants felt existing programs were effective. This implementation gap was from structural resource limitations, including shortages of trained staff and limited local funding. A gap appeared between the design of formal policies and feasibility in practices. Although female-focused sterilization has been recommended to enhance reproductive impact [11], [39], the approach may be difficult to implement in settings with substantial labor demands and chronic shortages of trained personnel. In certain settings, an opportunistic sterilization strategy involving the sterilization of all captured dogs irrespective of sex was regarded as a more operationally feasible approach under resource-constrained conditions. Sterilization also contributed to rabies prevention by reducing hormonal influences, thereby decreasing roaming and intra-pack aggression. Since rabies transmission primarily occurred through biting events, reductions in aggressive interactions reduced bite incidence and indirectly disrupted transmission dynamics within the dog population [40]. Meanwhile, there was strong support for enforcement against abandonment. Participants also proposed practical harm-reduction measures, recognizing that abandonment may not be entirely preventable. One suggestion was compulsory sterilization before abandonment. If ownership fails, the dog did not contribute to population growth. Although this approach may raise ethical and administrative concerns, the “sterilization-first” concept reflected a community perspective that prevention strategies should consider real-world behaviors rather than relying solely on ideal compliance.

The findings revealed a paradox in economic responsibility. Although 40.4% of respondents acknowledged municipal budget shortfalls, few supported direct co-funding (33.3% for full fees; 19.3% for co-payment). Because household economic constraints limit private contributions, formal cost-sharing is impractical. Sustainable rabies prevention must instead pivot toward non-monetary community participation to maintain local momentum under tight financial constraints.

Rabies control efforts were perceived as highly sensitive to political and administrative changes. NGO representatives emphasized that expanding collaboration could mitigate local resource constraints and regulatory barriers. Clearer partnerships are essential to resolve operational friction at the source and reduce misunderstandings between public health authorities, animal welfare groups, and the public. Effective policy implementation requires integrating multi-sectoral stakeholder perspectives with practical possibilities. In conclusion, policy implementation gap appears partly driven by limited legal enforcement and owner accountability. Although a pet registration system has been introduced, it remains in an early stage with relatively low public participation. Consequently, dog abandonment continues despite legal prohibitions. This gradually increases the free-roaming population and limits effective vaccination and sterilization programs. Instead of relying solely on reactive treatment after dog bites occur, policymakers may strengthen community engagement and multi-agency cooperation to achieve long-term rabies elimination.

The in-depth results revealed that fragmentation in sectoral perspectives contributed to institutional tension in rabies control strategy. Public sector described limited governmental support, particularly through insufficient service quotas that restricted access to vaccination and sterilization programs for cooperative owners. This situation appeared to generate community dissatisfaction. Meanwhile, NGOs raised concerns about the consistent enforcement of animal welfare standards. Government practitioners described a complex “double bind” situation. Although they recognized the epidemiological importance of controlling suspected rabid dogs during outbreaks, they often lacked clear legal authority and sufficient social support to act decisively. Tensions were perceived between public health law, which eliminates reservoir control during outbreaks, and animal welfare law, which restricts the killing of animals except under defined circumstances. In practice, rabies diagnosis requires laboratory confirmation by brain autopsy, resulting in uncertainty when responding to suspected cases. The lack of legal clarity may have exposed practitioners to criticism whether they acted or not.

Despite these differences, there was broad agreement across stakeholders that consistent sterilization and rabies vaccine campaigns were essential for effective dog population management and rabies control. The main challenge may not have been a lack of common goals, but rather the absence of a clear and unified operational framework. In this context, academic and research institutions can play a role by supporting dialogue and offering evidence-based guidance. This form of mediation may help reconcile animal welfare principles with public health priorities. Enhanced multi-sectoral coordination under a One Health framework could additionally provide a more integrated, evidence-driven approach, reducing conflict and contributing to more sustainable rabies control efforts. High rabies rates and ongoing demand for PEP show that rabies cannot be solved by public health or veterinary agencies alone. Because it is a system-wide problem, a One Health approach is essential to connect human, animal, and environmental sectors for sustainable disease control.

There were some limitations in this study. First, our study provided in-depth qualitative results, however, it might not be fully representative of the broader national population or regions with different cultural attitudes toward animal ownership and rabies awareness. Second, the data relied on self-reported perceptions and practices, which were susceptible to social desirability bias, particularly concerning sensitive topics such as animal abandonment, illegal culling, or individual willingness to pay for veterinary services. Third, while a legal paradox between public health legislation and animal-welfare laws was identified, this study did not include a formal legal audit. Further research is required to clarify the legal framework and incorporate legal experts' perspectives to help resolve these contradictions during active outbreaks. Fourth, while the study identified resource and budget constraints as primary barriers to sterilization, it did not include a longitudinal cost-benefit analysis comparing the long-term savings of aggressive sterilization against the recurring costs of reactive human PEP. In addition, primary facility-level PEP expenditures could not be specified due to varying hospital pricing structures and consent constraints for dog-bite incidences, further economic evaluations incorporating dynamic pricing data are needed to address this information gap and provide policymakers with a clearer financial framework for sustainable rabies elimination.

## 5 Conclusion

Progress toward rabies elimination may require shifting from fixed annual quotas to more flexible strategies that respond to local dog population dynamics. The study findings revealed that implementation challenges may result not only from resource limitations but also from regulatory complexity. Improved alignment between public health mandates and animal welfare legislation could help provide clearer operational guidance for practitioners. Strengthening coordination within One Health aspect, including recognizing the role of community members who informally care for free-roaming dogs, may help better integrating community practices with formal control programs. More consistent support for surveillance and preventive activities could help sustain program continuity beyond short-term administrative cycles. Proactive measures targeting dog population are essential for promoting sustainable rabies control. Multi-sector collaboration will also further support Thailand's advancement toward its national elimination goals.

## CRediT authorship contribution statement

**Weerakorn Thichumpa:** Writing – review & editing, Writing – original draft, Visualization, Validation, Supervision, Project administration, Methodology, Investigation, Formal analysis, Data curation, Conceptualization. **Anuwat Wiratsudakul:** Writing – review & editing, Validation, Resources, Methodology, Data curation, Conceptualization, Formal analysis, Supervision. **Wirichada Pan-ngum:** Writing – review & editing, Validation, Resources, Methodology, Data curation, Conceptualization. **Thomas Rawson:** Writing – review & editing, Validation, Methodology, Conceptualization. **Katharina D. Hauck:** Writing – review & editing, Validation, Supervision, Methodology, Conceptualization. **Tini Garske:** Writing – review & editing, Validation, Supervision, Methodology, Conceptualization.

## Ethical approval

This study was approved by the Ethic Committees of Faculty of Tropical Medicine, 10.13039/501100004156Mahidol University, Bangkok, Thailand (MUTM 2025-057-01).

## Submission declaration

• The article has not been previously published.
• The article is not under consideration for publication elsewhere.
• The article's publication is approved by all authors and tacitly or explicitly by the responsible authorities where the work was carried out.
• If accepted, the article will not be published elsewhere in the same form, in English or in any other language, including electronically, without the written consent of the copyright-holder.

## Funding statement

WT, TR, TG, and KH acknowledge funding from the Medical Research Council (MRC) Centre for Global Infectious Disease Analysis (MR/X020258/1) funded by the UK MRC and carried out in the frame of the Global Health EDCTP3 Joint Undertaking supported by the EU. TR and KH acknowledge funding from Community Jameel and Kenneth C Griffin supporting the work of the “Jameel Institute-Kenneth C Griffin Initiative for the Economics of Pandemic Preparedness” at the Jameel Institute for Disease and Emergency Analytics, Imperial. TR was supported by the Moh Family Foundation and the 10.13039/501100000275Leverhulme Trust (Grant RC-2018-003) for the Leverhulme Centre for Demographic Science. WP was supported in part, by the 10.13039/100010269Wellcome Trust [315982/Z/24/Z]. This study was also funded by the UK Government's International Science Partnerships Fund (ISPF) and delivered in partnership with the 10.13039/501100000308British Council, ISPF Early Career Fellowships in Epidemiology and Control of Infectious Diseases (Thailand) under the umbrella of MRC Centre for Global Infectious Disease Analysis (MRC GIDA), 10.13039/501100000761Imperial College London. The funders of the study had no role in the study design, data collection, data analysis, data interpretation, or writing of the report. For the purpose of open access, the authors have applied a Creative Commons Attribution (CC BY) licence to any Author Accepted Manuscript version arising from this submission.

## Declaration of competing interest

The authors have declared that no competing interests exist.

## Data Availability

Data will be made available on request.

## Appendix A Supplementary data

Supplementary material 1 Supplementary 1: Semi-structure interview form and questions.

Supplementary material 2 Supplementary 2: Summary of secondary data (annual estimates based on local reports, 2021–2025).
