# Supplementary material for: A mixed-methods study of community dog management and rabies awareness in Thailand
Source: One Health. 2026 Sep 28;23:101596. doi: 10.1016/j.onehlt.2026.101596 (PMC13659610; doi:10.1016/j.onehlt.2026.101596)
Supplement: Supplementary file 1 [file mmc1.docx]

**Supplementary information 1**

Semi-Structured Interview Form

**Section 1: Participant Background**

- Please tell about yourself (e.g., age, occupation, roles in the community, and other backgrounds)
- What is your roles or involvement with community dogs in your area?
- Do you own or feed dogs in community? If yes please share about your activities for dog caring.

**Section 2: Rabies awareness**

- What do you know about rabies disease and how it is transmitted?
- How serious do you think the problem of rabies is in your community or province?
- Have you or someone you know ever encountered a case of rabies in animals or humans? Please share your experience with rabies
- What actions do you take if someone is bitten by a dog? How do you recommend the practice for treatment and prevention?
- Please share your ideas for effective ways to prevent rabies
- Please share your experience with rabies campaigns and local interventions

**Section 3: Community Dogs**

- Are there community dogs (stray or free-roaming or partially owned dogs) in your area? If yes, please provide information and dog characteristics
- Who usually takes care of these dogs (e.g., owners, feeders)? How do they do?
- Where do these dogs usually stay or free roam?
- What is the general attitude of the community toward these dogs?
- In your opinion, do community dogs cause any issues or benefits in your area?
- What are the health benefits to pet dogs?
- Please share your opinion on pros/cons regarding community dogs
- Opinions on owner responsibilities without support budget from government

**Section 4: Interventions and Policy**

- Please share about rabies vaccination, dog sterilization, and prevention programs in your area
- Have you or your community participated in these programs? If yes, how?
- What challenges or successes have you seen with these programs?
- How do you think these interventions affect the dog population and community health?
- Any campaigns in community? And are there any impacts on rabies prevention and population control?
- Please share about animal and intervention services, including NGOs and private sectors
- Please share about the limitations of campaigns or related processes
- Please share about the regulation aspects

**Section 5: Cost Evaluation – Willingness to Pay and Cost-sharing**

- Do you think community members should contribute financially to rabies prevention (e.g., dog vaccination)? Please share your opinions
- Would you personally be willing to pay or co-pay for vaccination or sterilization of community dogs? How?
- What factors would influence your willingness to pay?
- In your opinion, who should be responsible for the costs of dog population control and rabies prevention aspects (government, owners/feeders, NGOs, or others?)
- What do you think about income may impact on costs of dog caring?
- How about any related treatment costs e.g. transportation, health follow-up

**Closing**

- Is there anything else you would like to share about community dogs or rabies prevention?
- Do you have any suggestions for improving dog-related programs in your community?
