# Supplementary material for: A mixed-methods study of community dog management and rabies awareness in Thailand
Source: One Health. 2026 Sep 28;23:101596. doi: 10.1016/j.onehlt.2026.101596 (PMC13659610; doi:10.1016/j.onehlt.2026.101596)
Supplement: Supplementary file 2 [file mmc2.docx]

**Supplementary information 2**

Summary of secondary data (annual estimates based on local reports, 2021–2025)

| **Information** | **Songkhla province** | **Chonburi province** | **References** |
| --- | --- | --- | --- |
| **Dog data** |  |  |  |
| Estimated dog population size | 90k-180k | 200k-420k | [1-6] |
| Estimated sterilized dogs | 4.5k-5k | 5k-8k | [1-6] |
| Estimated vaccinated dogs | 15k-23k (per district) | 9k-15k (per district) | [1-6] |
| Annual vaccination coverage in high endemic areas | >70% | >70% | [5, 6] |
|  |  |  |  |
| **Human data** |  |  |  |
| Annual human cases (range) | 0-2 | 0-2 | [3, 5, 6] |
| Estimated annual human PEP | >20k | >20k | [5-7] |

**References**

1.	Dog population reports. Rabies One Data.http://www.rabiesonedata.ku.ac.th/, 2025 (accessed December 2025).

2.	Thai rabies report. Thai Rabies Net.http://www.thairabies.net/trn/, 2025 (accessed 15 January 2026).

3.	Rabies Situation in Thailand, 2024–2025. Division of Epidemiology, Department of Disease Control, Ministry of Public Health.https://he05.tci-thaijo.org/index.php/WESR/article/view/5568/5121, 2025 (accessed 1 October 2025).

4.	Rabies situation report in animals. Department of Livestock Development, Ministry of Agriculture and Cooperatives.https://dld.go.th/th/index.php/th/64-hot-issue/rabies, 2025 (accessed 15 November 2025).

5.	Local reports of dog rabies and human PEP. Songkhla Provincial Livestock Office.https://region9.dld.go.th/index.php/th/home-office4, 2025 (accessed 18 January 2026).

6.	Local reports of dog rabies and human PEP. Chonburi provincial livestock office.https://region2.dld.go.th/index.php/th/offices/cbr, 2025 (accessed 20 January 2026).

7.	Registry of contacts exposed to rabies‑positive animals (in Thai language). Department of Disease Control, Ministry of Public Health.https://opendata.ddc.moph.go.th/th/organization/odpc12, 2025 (accessed 19 January 2026).
